# Enumeration of microparticles on a gridded filter using a stratified random sampling tool

**DOI:** 10.1016/j.mex.2023.102284

**Published:** 2023-07-08

**Authors:** Kayli Paterson, Michael Silverstan, Barbara Beckingham

**Affiliations:** a Environmental & Sustainability Studies Program, College of Charleston, Charleston, SC, United States; b Oregon State University in Corvallis, OR, United States

**Keywords:** Stratified random sampling, Particle enumeration, Microplastics, Microparticles, Tire particles, Filter, Microscopy, Stereomicroscopy, Particle distribution, Patterned distribution, *Method for the enumeration of microparticles on a gridded filter using a stratified random sampling tool*

## Abstract

Quantifying microplastics and other microparticles is a matter of interest in the field of environmental science. Stereomicroscopy is one of the most common methods to identify and enumerate micro-size particles. However, the process of enumerating an entire environmental sample can be tedious and time-consuming, especially when target particles are abundant. Here we present a method to develop a subsampling strategy and spreadsheet-based tool to speed up the process of microparticle enumeration while maintaining particle count accuracy. We first identified the pattern in which tire road wear particles (TRWPs) from environmental samples were distributed on a filter when vacuum-plated, then used particle abundance within relatively homogeneous subsection arrangements to establish stratified random subsampling schemes. We describe a repeated sampling experiment using count data to test the stratified design and illustrate the relationship between the fraction of the filter counted (sample size) with accuracy and variance in the extrapolated total sample count and the corresponding analyst time savings when applied to analyzing TRWPs isolated from sediments. Based on the results, a particle enumeration tool was created in Microsoft Excel Visual Basic^Ⓡ^ configured using a 47 mm gridded filter, and the source is available for free modification under the same open license.

• Vacuum-plated microparticles are often highly abundant and not homogenously distributed across a filter.
• A random sampling selection data tool was created using knowledge of particle distribution.
• Method describes how to structure and use partial filter counts to extrapolate for total particle enumeration.

## Graphical abstract

**Figure:**
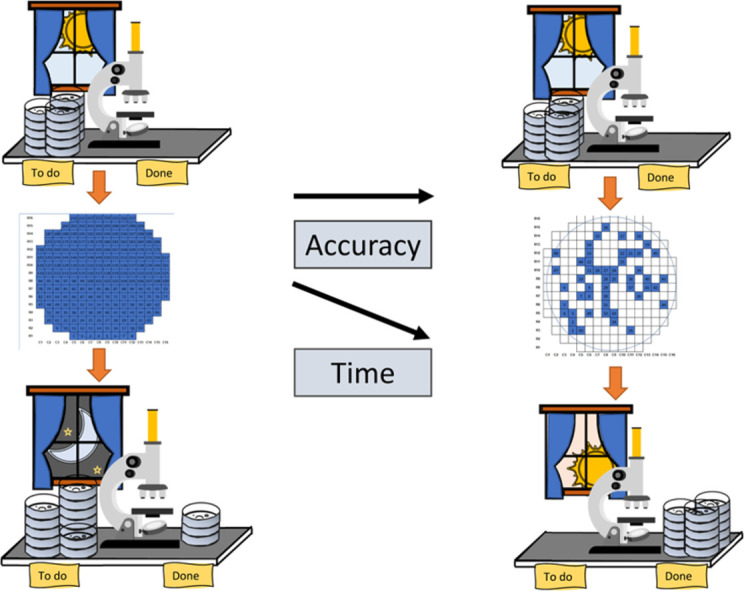

Specification table

**Table:** 

Subject area:	Environmental Science
More specific subject area:	Statistical subsampling for particle enumeration
Name of your method:	Method for the enumeration of microparticles on a gridded filter using a stratified random sampling tool
Name and reference of original method:	*n/a*
Resource availability:	*https://github.com/KayliPaterson/SamplingTool*

## Method details

### Background

The identification and enumeration of micro-sized particles such as microplastics or tire wear particles in environmental matrices using microscopy can be highly time-consuming due to the need to inspect each particle in a field of vision that can include other non-target particles. Visual identification and quantification of particle abundance using counts is a common practice, even if followed by additional material characterization such as spectroscopy, since the method is non-destructive and the equipment is relatively accessible and inexpensive [[2], [8]]. Large sample volumes (i.e., soil, sediment, water) and particle number may need to be analyzed for representativeness of microparticle characterization considering concentration and variation within the matrix [1]. However excessive time scrutinizing particles through an eyepiece can lead to analyst fatigue and burnout. For example, a study by [4] found that the detection probability of some types of micro- and meso‑plastic debris (2.5–60 mm) by observers decreased during a trial lasting ∼1.5 hrs in which observers recorded abundance in shoreline quadrats, showing fatigue can play an important role in accurate microplastic detection. Machine learning methods to count and classify microplastics are a promising approach but are still in development [7]. Machine learning may underperform if tactile information is valuable for microparticle identification, which is often the case for microplastics and tire wear particles [3,9]. Thus, although microscopy equipment is commonly available to researchers, the labor-intensive steps of counting and isolating suspected microplastics from samples can make large-scale monitoring studies time or cost-prohibitive, so there is a need for improved subsampling methods [6]. Therefore, we developed a randomized subsampling methodology and tool to improve the efficiency of particle enumeration in the visual identification process. This tool extrapolates the particle counts on a fraction of the filter to the entire filter while retaining accuracy based on the location probability of particles when vacuum-plated on a gridded filter.

This method presents how to develop a subsampling strategy and spreadsheet-based tool to aid the enumeration of tire road wear particles (TRWPs) that are isolated imperfectly from an environmental matrix. However, the method may be applied to quantify any particle type, and the spreadsheet-based tool can be easily modified to complement different microparticle studies. At the final stage of sample preparation for microscopy, the collected particles from an environmental sample were washed into a vacuum apparatus with a filter flask containing a gridded filter. We used a 47 mm diameter, mixed cellulose membrane filter, pore size 0.45 µm, with 3.1 mm grid cells (Millipore Ez-Pak or Whatman/Cytiva). During the plating process, care was taken to ensure the particle distribution across the filter was as even as possible. This was done by rinsing the sides of the funnel flask and resuspending the particles using a gentle stream of filtered DI water from a glass pipette. Then, once most of the particles were suspended, the vacuum suction was engaged to remove the water. Even so, and as reported by others, microparticles do not settle homogenously on a filter, and a statistical design to extrapolate a subsample to a total population count needs to be informed by the spatial distribution of particles [10]. Therefore, after the particles from a set of environmental samples were plated, TRWPs were enumerated using visual and tactile identification [3] to 1) determine the distribution of tire particles on the filter, 2) estimate the accuracy and time needed to analyze different proportions of the total filter, and 3) streamline the counting analysis by developing an interactive stratified random sampling tool using Visual Basic for Applications (VBA 7.1) in Microsoft Excel (Version 2301).

### Testing the distribution and homogeneity of vacuum-plated particles

**Fig. 1:**
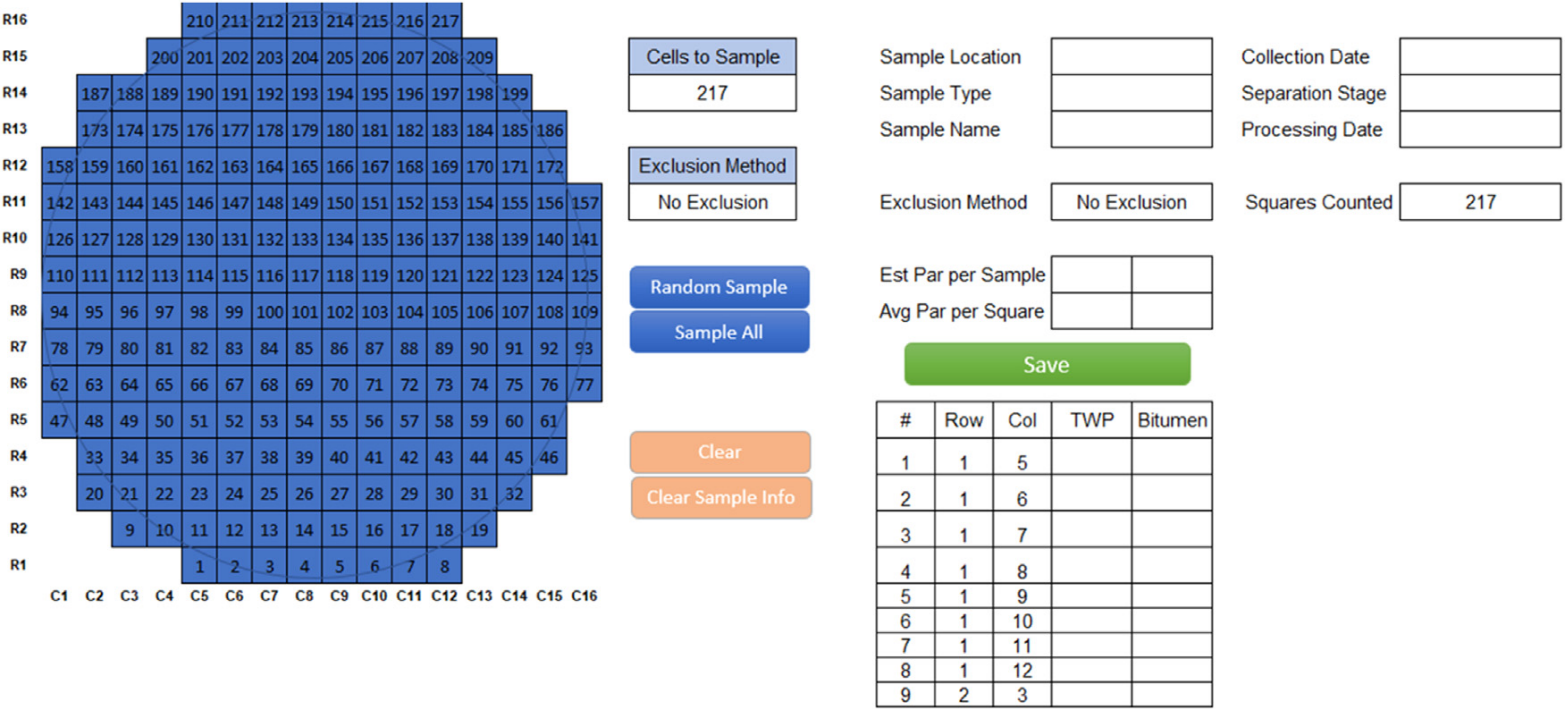
Lay out of the filter counting program utility in Microsoft Excel Visual Basic. Colored buttons are functions, filled grid cells indicate the selection to count (in this example, the entire filter is selected with no exclusion), empty grid cells show where information and results are either inputted (e.g., sample information at the top-right, and count data for tire road wear particles (here denoted as TWP) and bitumen in bottom table) or returned (estimated particles (Par) per sample or average per square in the center of layout). The gray circle on the filter indicates the placement of the filter funnel and marks the outline of the exposed filter area.

The first step to developing a spatial subsampling tool to estimate the total number of microparticles on a filter is to understand how the variable of interest (here, microparticle counts) is distributed across the finite population of sampling units (here, grid cells among a total number on a 47 mm diameter filter, *N* = 217 or with partial grid cells excluded *N* = 167) and whether the random sampling arrangement should be simple (full randomization across the domain) or stratified (randomized within homogenous sections, or strata). The location distribution of filtered particles was profiled following previously published work by Thaysen et al. [10], who also used a vacuum-filter apparatus to plate microplastic particles from water samples onto a gridded filter. To profile the particle distribution in the current study, five environmental sediment samples (sieved between 63 and 500 µm) containing TRWPs were prepared for microscopy analysis and plated on the gridded filter described above. Every grid cell sampling unit on the filter was given a grid cell ID number, and the arrangement of numbered cells was programmed in Excel Visual Basic^Ⓡ^ (Fig. 1). Particles were identified under a stereomicroscope, with the number of particles counted on each filter cell input into the corresponding grid cell ID in the program utility. The process of identifying the TRWPs in the environmental samples using visual and physical attributes was timed so time savings could be evaluated.

**Fig. 2:**
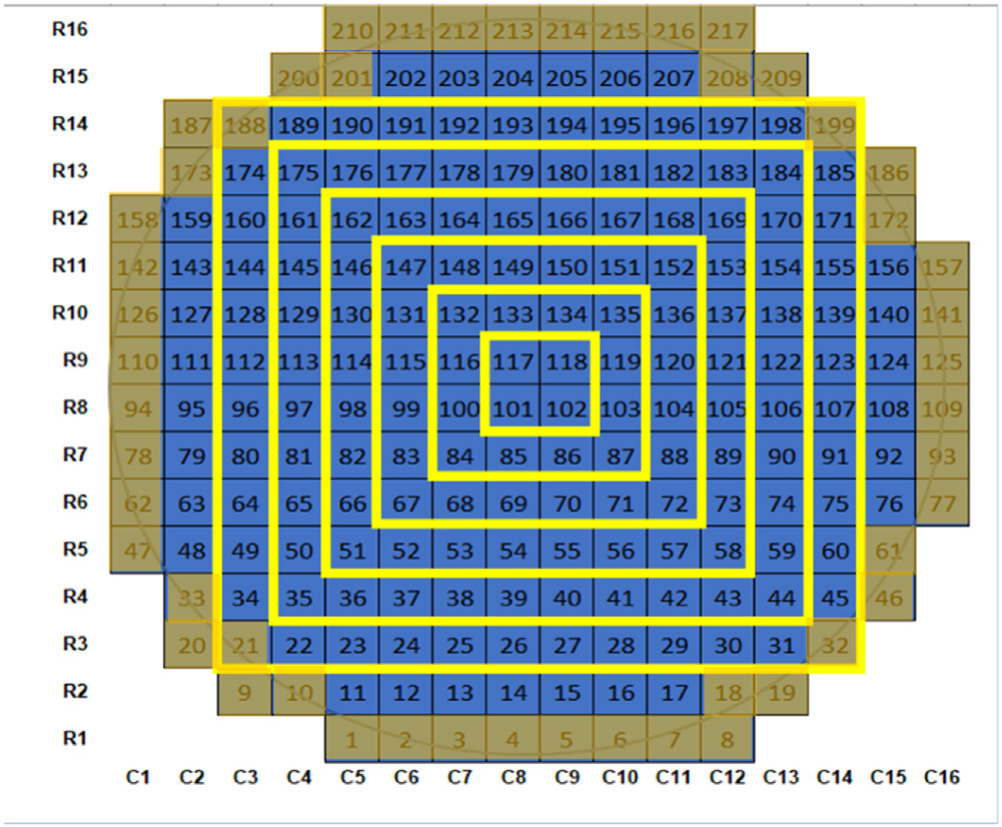
Subsections defined on the gridded filter with numbered rows (16), columns (16), and grid cells (217) with the “bullseye” pattern of concentric rings (yellow lines) where sections 1 (center) through 7 (outside) are defined by the distance from the center, and partial grid cells (brown) removed results in a lower number of grid cells (167).

**Fig. 3:**
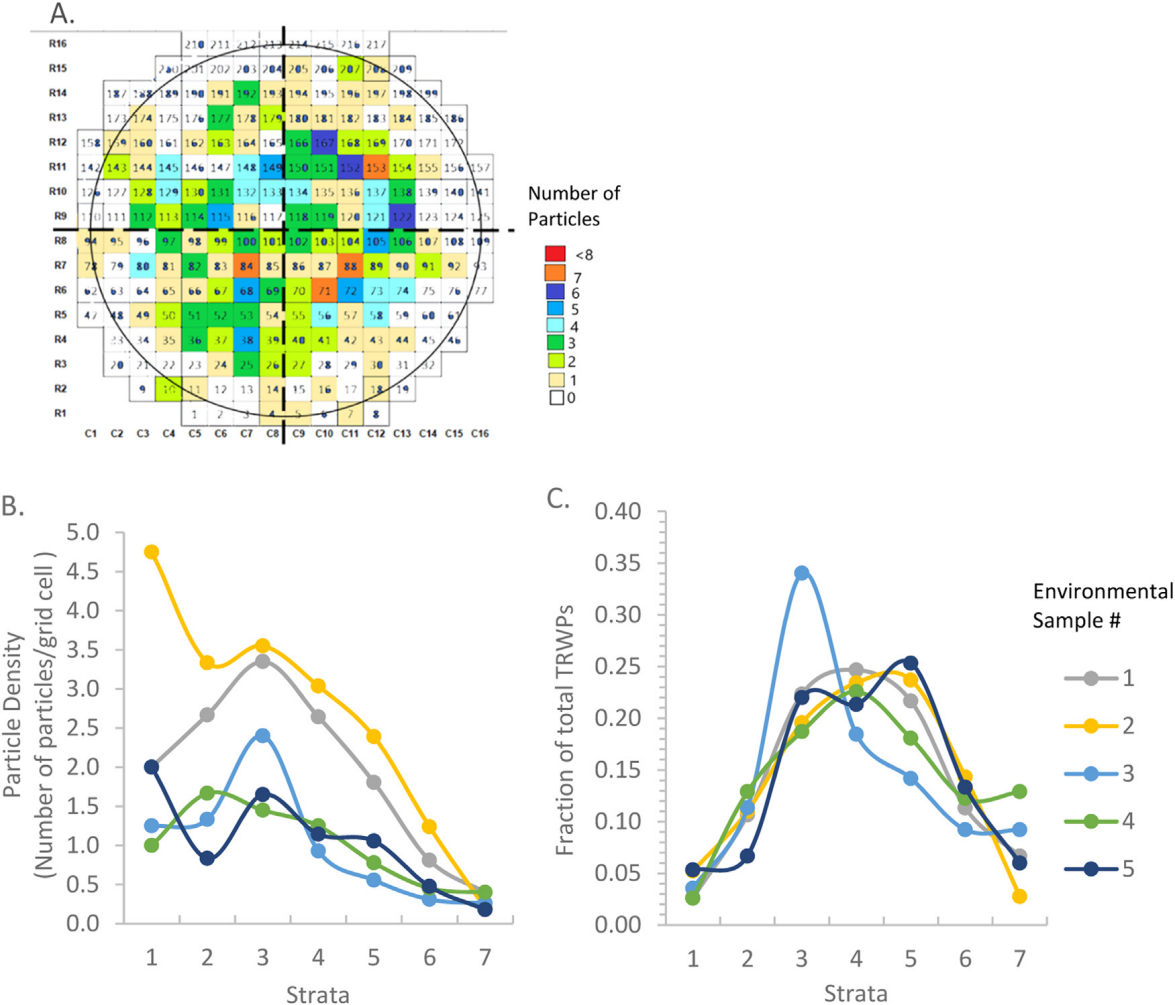
Distribution of TRWPs across bullseye strata on the filter is illustrated using A. heat maps, such as this example for environmental sample #1, B. particle density (average number of TRWPs/grid cell, with partial cells in strata 6 & 7 counted as 0.5 cells) and C. fractional TRWPs counts (count in strata/total TRWP count) for each environmental sample (1–5).

We used the starting point provided by Thaysen et al. [10] who observed that vacuum-filtered microplastics were arranged in a “bullseye” pattern, with higher density near the center of the filter. We divided the filter into seven strata in this concentric ring pattern (Fig. 2) and confirmed that our filtration technique showed a similar pattern by visualizing the distribution using heat maps and filter cross-sectional profiles of particle counts and densities (Fig. 3). In the example heat map, we can observe that the distribution is not homogeneous and that there is a tendency for particle density to be centralized (Fig. 3A). The particle density profiles show a distinct, repeatable pattern of particle plating, with the subsections towards the center of the filter (1–3) having a higher particle density than the subsections near the outside of the filter (6–7) (Fig. 3B). Particles were more abundant in the midsection, given a larger number of grid cells in those strata (3–5; Fig. 3C). The average percentage of particles per subsection (*N* = 5) from the center to the outside, subsections 1 through 7, was 4.0% ± 1.2%, 10.8% ± 2.2%, 24.0% ± 5.8%, 22.7% ± 2.1%, 21.1% ± 4.0%, 12.4% ± 1.7%, and 5.1% ± 2.7%, respectively.

Homogeneity of particle grid cell counts across the entire filter and within each concentric ring section was evaluated using the coefficient of variation (CV). The coefficient of variation (CV) is used to compare the relative amounts of variation in populations having different means. CV is calculated by dividing the standard deviation (δ) by the mean ($\bar{Y}$) of the number of particles per grid cell and multiplying it by 100 to create a percentage, shown in Eq. (1), with homogeneous distribution generally assigned to a CV less than 10%.

(1) $$CV=\frac{\delta }{\bar{Y}}*100$$

Results are shown in Table 1, with the outermost ring (#7) evaluated with and without the edge grid cells excluded. The filter holder obstructed these partial cells, resulting in low particle counts (Fig. 2). For instance, excluding the outermost partial cells reduces the number of grid cells to count to *N* = 167, a reduction of 23%, and greatly reduced variance in the outer strata (#7, Table 1). However, the total known count of TRWPs was only reduced by 0–3.5%. CV for the whole filter varied between 120%−178%. Subsections 1–5 on the “bullseye” pattern have lower CVs than for simple random sampling of the entire filter, showing that the distribution of TRWPs is more homogeneous in the central part of the filter and that adopting a bullseye stratified sampling scheme could improve estimation efficiency. While an improvement overall, there was still relatively high variation around the outside of the filter (subsections 6–7). Higher CV in the outer sections of the filter corresponds to lower particle density (Fig. 3B).

**Table 1:** Coefficient of variation (CV%) for subsections in the “bullseye” pattern.

Environmental Sample #	Entire filter	Grid subsection (per pattern in Fig. 2)							
		1	2	3	4	5	6	7	7-no partials
1	120	61	67	60	63	79	132	194	153
2	124	50	69	57	62	89	121	304	199
3	178	35	88	81	122	172	223	284	240
4	142	71	79	91	87	122	181	220	150
5	149	94	96	75	80	124	159	267	187

The next step is to optimally group subsections and apportion sample size to find a simplified, efficient stratified random sampling design. The principle of stratification is that estimation of the total particle counts will be more precise if the population of grid cells is partitioned into strata so that within each stratum, the particle counts per grid cell are as similar as possible (i.e., lower variance). We used two approaches to select two different strata arrangements for model comparison: 1) cluster analysis and 2) analysis of the coefficient of variance and particle distribution.

**Fig. 4:**
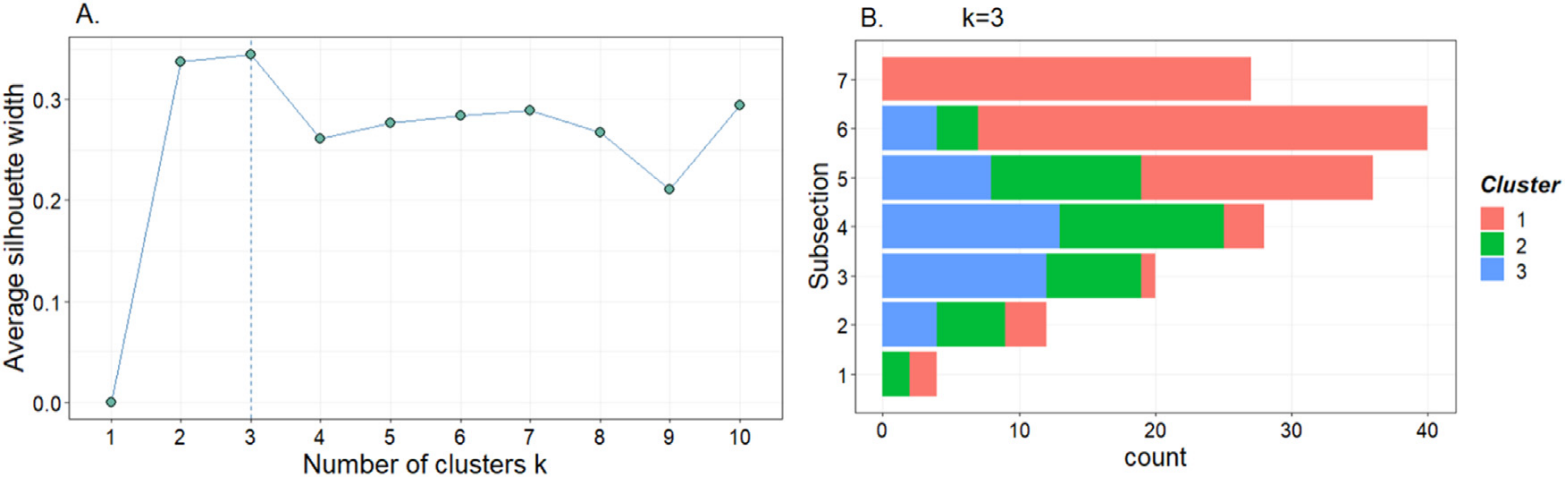
Analysis of the optimal strata arrangement using *k-means* cluster analysis. A) The optimal number of clusters using the average silhouette method, B) a *k* = 3 cluster analysis grouping the observations from each subsection into the assigned cluster.

As a first step to identify the optimal strata arrangements, a cluster analysis of the raw tire particle count data was performed using *k-means* analysis in RStudio (Version 2022.12.0). The *k-*means clustering algorithm has broad applicability in clustering applications and is an iterative algorithm that finds locally optimal solutions with respect to the clustering error [5]. It does this by partitioning observations into a pre-specified number of clusters. An average silhouette static for *k-means* was performed on the raw data to find the optimal number of clusters for the *k-means* cluster analysis. As shown in Fig. 4A, the ideal number of clusters for our *k-means* cluster analysis is three. The cluster analysis identified several suitable subsection arrangements using three as the pre-specified number of clusters. Isolating the best-performing cluster arrangement was difficult due to substantial overlap between the clusters. Variance analysis was conducted from the *k-means* clustering algorithm to illustrate how subsections fit into different clusters, as shown in Fig. 4B. Based on the *k-means* cluster analysis (Fig 4), we defined three strata (h_1–3_) as groupings of subsections, from center, with [3:2:2] where h_1_= 1–3, h_2_ = 4–5, and h_3_ = 6–7.

In addition to this cluster analysis approach, we calculated the CV of selected subsection groupings based on observed particle density distribution (Fig. 3, Table 2) to aid selection. We compared the variance in the stratified cell counts to the variance in the entire filter with no partial cells in Table 2 [12]. The groupings with the subsections near the center – (1–2), (1–3), and (3–4) – had the lowest amount of variation. While the variation in the subsection groupings further from the center (5–7) and (6–7) had higher variation, most likely driven by subsection 7. The interaction between the filter and the funnel flask most likely causes the considerable variation in subsection 7. Considering particle distribution and variance in groupings, the second stratified random sampling scheme with three strata grouping subsection was chosen as [3:3:1] where h_1_ = 1–3, h_2_ = 4–6, and h_3_ = 7.

**Table 2:** Variance (CV%) in particle counts per grid cell for example strata groupings of concentric ring subsections.

Sample	CV% for subsection groupings								
	Full	1–2	1–3	4–5	4–6	3–4	5–7	6–7	7-no partials
1	99	68	65	74	93	63	117	137	153
2	99	65	61	77	92	60	127	145	199
3	155	80	88	146	167	109	202	222	240
4	120	82	87	105	125	90	145	161	150
5	125	113	90	106	122	80	159	166	187
Average	120	82	78	102	120	80	150	166	186

In stratified sampling, the next step is to determine the method for allocating sample size among strata. There are different allocation schemes [11], but we chose optimum allocation that allocates based on producing the lowest variance in the total count. We calculated the number of grid cells to count in each stratum of the 3:3:1 and 3:2:2 stratified arrangements using Eq. 2

(2) $$\frac{n_{hi}}{n}=\;\frac{N_{hi}\sigma_{hi}}{\sum_{h=1}^{H}N_{h}\sigma_{h}}$$

where $n_{hi}$ is the sample number to allocate to the ith strata, *n* is the total desired sample size (i.e., number of grid cells to count), $N_{hi}$ is the number of sampling units, i.e., grid cells comprising the ith strata, and $\sigma_{hi}$ is the standard deviation in particle count per grid cell. The average results across the five environmental samples were simplified to an allocation scheme of $\frac{n_{hi}}{n}\;\times 100\%$ in *h_1_, h_2_*, and *h_3_* of 30%, 60%, and 10% for the 3:3:1 scheme and 30%, 40%, and 30% for the 3:2:2 scheme, respectively. Likely because variance was related to particle density, we find that this optimum allocation by variance was similar to proportional allocation on the basis of particle counts (Fig. 3C, Table 3). Allocation on the basis of cost, i.e., sampling time, to improve efficiency could also be done, however, we did not pursue this due to lack of data on sampling time per grid cell in the different strata. This could vary as a function of particle density but also abundance of interfering background material, and that the overhead time cost associated with repositioning and focusing the filter under the microscope would also need to be accounted.

**Table 3:** Example sample allocation based on lowest variance (Eq. (3)) or particle abundance (% of total particle count within strata) for two strata arrangements.

**Strata**	**Percent of total grid cells to count in each strata by scheme**					
	**3:3:1**			**3:2:2**		
	*Subsection Groupings*	*Lowest variance*	*Particle abundance*	*Subsection Groupings*	*Lowest variance*	*Particle abundance*
**h_1_**	1–3	30	29	1–3	30	29
**h_2_**	4–6	60	63	4–5	40	42
**h_3_**	7	10	8	6–7	30	36

### Evaluating random sampling schemes

The objective was to identify a sampling design (simple random or stratified random) with strata sampling weights and the smallest sample size needed to consistently produce accurate total particle counts. We performed a stochastic evaluation of the selected stratified sampling arrangement (3:3:1 with 30%:60%:10% allocation and 3:2:2 with 30%:40%:30% allocation) with and without partial cells (Fig. 2) compared to simple random sampling by estimating total particle count on the filter with different sample sizes or proportions of the filter counted: 10 cells (5% of the total, 6% excluding partials), 30 cells (14% of the total, 18% excluding partials), 50 cells (23% of the total, 30% excluding partials), 80 cells (37% of the total, 48% excluding partials), and 110 cells (50% of the total, 66% excluding partials) to validate our method.

The sampling arrangements were evaluated by creating a randomization program in Microsoft Excel^Ⓡ^ and using the data collected from the five environmental samples to test the accuracy of each stratification scheme and filter portion counted. Once the unique grid cell ID numbers were assigned to their “bullseye” subsection boxes and the cell counts entered, the VLOOKUP function in Microsoft Excel^Ⓡ^ could reference the data using cell ID, the INDEX function to reference the proportion of cells to sample in each subsection and the RANDBETWEEN function to scramble and pick out the random cell IDs needed from the designated subsections to count the total number of desired cells (e.g., 10, 30, 50, 80 or 110 squares, corresponding from ∼5% to 50% of the finite population of grid cells on the filter or ∼6% to 66% when partial grid cells are excluded). The parameter estimate, total number of TRWPs in an environmental sample plated on a filter, was calculated according to Eqs. (3) and (4),

(3) $$\tau =\sum_{h=1}^{H}\frac{\tau_{h}}{f_{h}}$$

(4) $$\tau_{h}=\frac{N_{h}}{n_{h}}\sum_{i=1}^{n_{h}}y_{hi}$$

where τ is the total count of TRWPs on the entire filter, which is the sum of all strata counts (*h* = 1, 2…H) from 1 (entire filter randomized) to *H* = 3 (according to stratified schemes), with τ*_h_* being the total count of TRWPs within a strata, *h*, that is the sum of $y_{hi}$, the count of TRWPs in the sample of grid cells of sample size $n_{h}$, scaled up by the sampled fraction of the total grid cells in that strata (*N_h_*) [11]. The sum of the number of grid cells in each stratum equals the entire population of grid cells in the filter domain. The stochastic simulation was performed with the stratified random sampling models run multiple times (20x) for each sample size, and the results compared the precisely known τ. We illustrate accuracy (percentage of estimates within 15% of the true known value) and variance in the resulting total particle count estimates for an environmental sample. Relationships between variance in the parameter estimate and cost (time spent based on the fraction of the filter counted) are also evaluated.

**Fig. 5:**
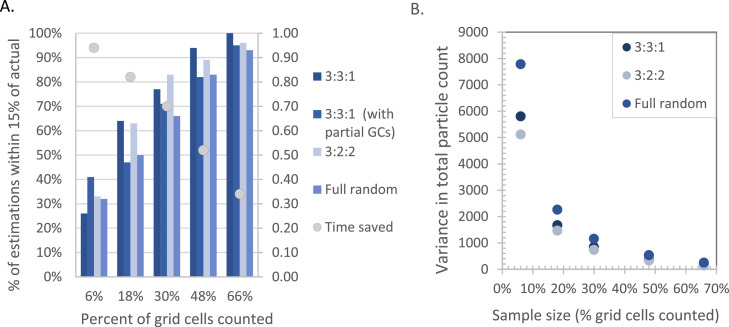
A. Percentage of total TRWP count estimations (τ) within ±15% of the actual TRWP count and the time saved by counting the subsample of grid cells and B) Variance in random sampling model estimates of total particle count for different sample sizes for an example Environmental Sample #1 with partial grid cells (GCs) excluded for all but 3:3:1 with results shown both with and without partial GCs.

Fig. 5A shows the percentage of trials for the 3:3:1 (with and without partial grid cells (GCs)), 3:2:2, and full randomization arrangements for which total TRWP count estimates (τ) fell within ±15% of the actual count number plotted with time saved against the fraction of total grid cells counted. Partial cells were excluded due to the previously described edge effects in plating and to avoid assigning a fractional grid cell area to partial squares when scaling up to total TRWP counts (τ). As predicted by statistical theory, estimations improve when a greater proportion of the total number of grid cells are counted (Fig. 5). Counting 66% of the grid cells (110) was consistently within ±15% of the actual value for the 3:3:1 arrangement (Fig. 5A). Estimated total counts tend to cluster closer to the true value when partial cells along the filter edge are excluded, compared to when they are included (see the comparison of 3:3:1 and 3:3:1 with partial GCs in Fig. 5A). The precision of the stratified subsampling schemes can further be compared using the relationship between sample size and variance in τ (Eq. (5), Fig. 5B) [11]. Both metrics demonstrate improved estimation by stratified random sampling in comparison to full simple random sampling (Fig. 5).

(5) $$\text{var}\left(\tau \right)=\sum_{h=1}^{H}N_{h}\left(N_{h}-n_{h}\right)\frac{\;\sigma_{hi}^{2}}{n_{h}}$$

The stratified random sampling tool is helpful because it increases analysis efficiency for samples with high particle abundance. The average counting rate was 88±21 min per filter (*N* = 5), about 0.4 min per grid cell, or 2.5 ± 0.6 TRWPs per minute based on the timed counting of the entire filter for several high-particle load samples (Environmental Samples 1–5). This average includes physical probing that could vary for other sites depending on the amount of organic matter and the number of particles. Fig. 5A shows the time savings based on the reduction in the number of grid cells analyzed. For example, counting particles for 80 grid cells would take ∼32 min, or half the time of analyzing the entire filter, while producing an estimate within 15% of the true value over 95% of the time.

The present work treats tire road wear particles as a single population on the filter. If multiple types of particles are targeted for enumeration, sample size (number of grid cells to count) and stratification scheme should consider the expected heterogeneity of particles by type. For example, studies typically classify microplastic particles into several types, such as fragments, films, foams, fibers, and spheres. Count accuracy could vary by type in particle mixtures. In general, a larger sample size would be required for more diverse classifications schemes and for finding “rare” types.

### Description of the stratified random sampling tool

**Fig. 6:**
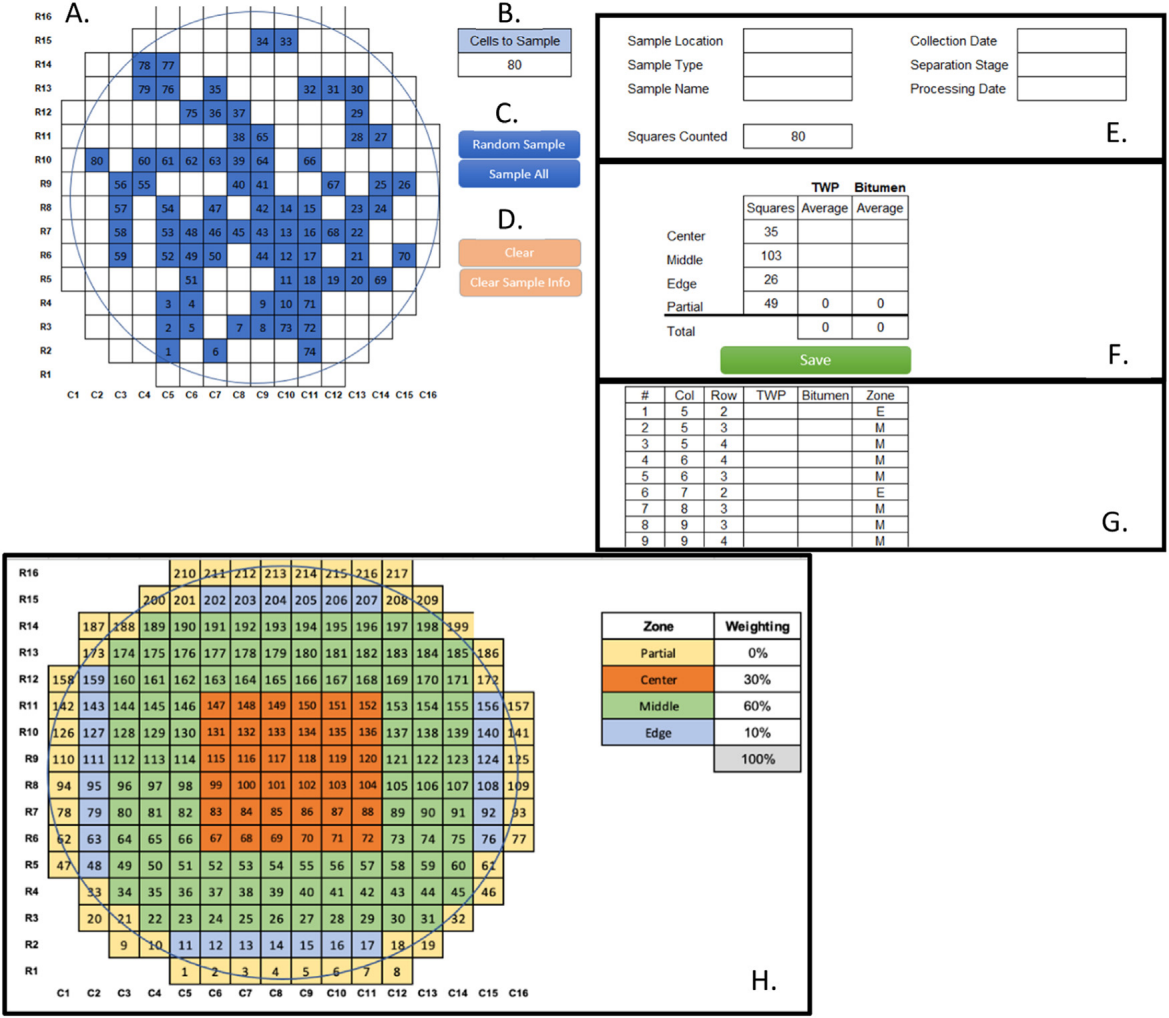
Example view of the Excel Visual Basic^Ⓡ^ 3:3:1-no partials grid cells stratified random sampling program. Different sections of the visual display are indicated by letters and/or boxes, with A-G on the “Calculator” tab and H on the Settings tab.

Based on the sampling scheme development in this study, a stratified randomization calculator tool using the Macro extension in Microsoft Excel (Version 2301) and programmed using Visual Basic for Applications (VBA 7.1) is presented to assist with microparticle counting. The final version of the program layout shown in Fig. 6 is a modification of the basic counting program (Fig. 1) with the addition of the stratified subsections as “center,” “middle,” and “edge” zones (C, M, and E in panel G, respectively). The total number of squares in each zone is shown in Panel F, and this counting program uses the 3:3:1 “no partial grid cells” scheme as the default for the stratified random sampling model. This option may be selected when plating produces a border effect where these grid cells are not a very useful sampling area due to high variance and low particle density. Otherwise, the tool has a “no exclusions” option, whereas these grid cells are included. Furthermore, section weights can be modified in the “Settings” tab (Fig. 6H).

The program displays where the sampled grid cells are located on the filter (Fig. 6A). The desired number of grid cells counted can be set in the "Cells to Sample'' box (Fig. 6B). When the number of cells to sample has been set, clicking the “Random Sample” button will sample the desired number of cells, while clicking the “Sample All” button will override the value in the “Cells to Sample” box, and all cells except the partial cells will be active (Fig. 6C). After the grid cells are selected, the utility runs the list through a simple pathing algorithm that starts at the bottom left origin, finds the next nearest grid cell, and lists them sequentially in the table (Fig. 6G). This arrangement creates a path that reduces unnecessary stage travel during sample counting and easy data entry. The “Clear” button will clear the random sample from the display, and the "Clear Sample Info'' will clear all information on the page (Fig. 6D). The Fig. 6E box contains entries for sample information and progress on the number of squares counted (i.e., cells with data input in panel G) that are automatically updated. The Fig. 6F box shows the number of TWPs weighted for the total section area used in the 3:3:1 model; this will be updated as more cells are counted. The “Save” button transfers the grid cell sample locations and count data to the “Data” tab (Fig. 6F). For our project, counts were collected on two different particle types, so there are two columns provided for data input for each designated cell in the Fig. 6G box. The type or name of surveyed particles can be updated as desired to fit a microparticle study. The VBA particle counting tool is freely available at our github portal: https://github.com/KayliPaterson/SamplingTool. The tool and spreadsheet are easily accessible and can be modified to suit different microparticle studies.

## Summary

This method describes how to evaluate the distribution of particles on a filter, establish stratified subsampling weights based on particle abundance and clustering, and offers a simple tool that can be adapted, if necessary, using Visual Basic. The tool is designed to randomly select a subsample within a pre-defined filter grid for particle analysis and make the data input and management easier for the user. The program allows cells within the filter grid to be assigned to a zone and weighting for that zone to be adjusted. This is a benefit if the assay conditions or equipment do not evenly distribute the particles across the filter, such as seen in our preparatory method, or if the exclusion of partial squares is desired. We established this method by targeting the enumeration of tire road wear particles from road dust samples and base the initial configuration on a specific 47 mm diameter filter with 3.1 mm grid cells, but the source code is available for free modification under GNU General Public License v3.0. Any modifications and reproductions of this program are required to be followed under the same license. After this method is used to set-up the stratified random sampling tool for the microparticles and laboratory system of interest, we expect that application of this tool for microparticle enumeration can reduce analysis time for the benefit of environmental research.

## Ethics statements

This protocol does not include human or animal subjects.

## Funding

This work was supported by South Carolina Sea Grant Consortium Award #R/ER-52.

## CRediT authorship contribution statement

**Kayli Paterson:** Conceptualization, Methodology, Investigation, Formal analysis, Writing – original draft, Data curation. **Michael Silverstan:** Methodology, Software. **Barbara Beckingham:** Conceptualization, Methodology, Funding acquisition, Writing – review & editing.

## Declaration of Competing Interest

The authors declare that they have no known competing financial interests or personal relationships that could have appeared to influence the work reported in this paper.

## Data Availability

Data will be made available on request.

## Appendix Supplementary materials

Image, application 1
