# Supplementary material for: Enumeration of microparticles on a gridded filter using a stratified random sampling tool
Source: MethodsX. 2023 Jul 8;11:102284. doi: 10.1016/j.mex.2023.102284 (PMC10407279; doi:10.1016/j.mex.2023.102284)
Supplement: Supplementary file 1 [file mmc1.docx]

**Supplemental Information**

**Table S1. Raw data of tire road wear particle counts for environmental samples 1-5**

| **Sample Number** | | **1** | **2** | **3** | **4** | **5** |
| --- | --- | --- | --- | --- | --- | --- |
| Total Number of Particles | | 300 | 363 | 141 | 155 | 150 |
| Average number of particles per grid cell | | 1.382 | 1.673 | 0.650 | 0.714 | 0.691 |
| **Grid cell ID** | **Location** | **Number of TRWPs per grid cell** | | | | |
| 1 | R01C05 | 0 | 0 | 0 | 0 | 0 |
| 2 | R01C06 | 0 | 0 | 0 | 0 | 0 |
| 3 | R01C07 | 0 | 0 | 0 | 0 | 0 |
| 4 | R01C08 | 1 | 0 | 1 | 0 | 0 |
| 5 | R01C09 | 1 | 0 | 0 | 0 | 0 |
| 6 | R01C10 | 0 | 0 | 0 | 0 | 0 |
| 7 | R01C11 | 1 | 0 | 0 | 0 | 0 |
| 8 | R01C12 | 0 | 0 | 0 | 0 | 0 |
| 9 | R02C03 | 0 | 0 | 0 | 0 | 0 |
| 10 | R02C04 | 2 | 0 | 0 | 0 | 0 |
| 11 | R02C05 | 1 | 1 | 0 | 0 | 0 |
| 12 | R02C06 | 0 | 0 | 0 | 1 | 1 |
| 13 | R02C07 | 0 | 0 | 0 | 0 | 0 |
| 14 | R02C08 | 1 | 0 | 0 | 0 | 0 |
| 15 | R02C09 | 0 | 2 | 0 | 0 | 0 |
| 16 | R02C10 | 1 | 0 | 1 | 0 | 0 |
| 17 | R02C11 | 0 | 0 | 3 | 0 | 0 |
| 18 | R02C12 | 1 | 0 | 1 | 0 | 0 |
| 19 | R02C13 | 0 | 0 | 0 | 0 | 0 |
| 20 | R03C02 | 0 | 0 | 0 | 0 | 0 |
| 21 | R03C03 | 0 | 0 | 0 | 0 | 0 |
| 22 | R03C04 | 0 | 1 | 0 | 3 | 1 |
| 23 | R03C05 | 0 | 1 | 0 | 0 | 0 |
| 24 | R03C06 | 1 | 1 | 1 | 2 | 0 |
| 25 | R03C07 | 3 | 1 | 0 | 0 | 1 |
| 26 | R03C08 | 2 | 3 | 1 | 1 | 0 |
| 27 | R03C09 | 2 | 2 | 1 | 1 | 1 |
| 28 | R03C10 | 0 | 0 | 0 | 1 | 1 |
| 29 | R03C11 | 0 | 1 | 0 | 1 | 0 |
| 30 | R03C12 | 1 | 0 | 0 | 0 | 0 |
| 31 | R03C13 | 0 | 0 | 0 | 0 | 0 |
| 32 | R03C14 | 0 | 0 | 0 | 0 | 0 |
| 33 | R04C02 | 0 | 0 | 0 | 0 | 0 |
| 34 | R04C03 | 0 | 0 | 2 | 0 | 0 |
| 35 | R04C04 | 1 | 0 | 0 | 0 | 0 |
| 36 | R04C05 | 2 | 1 | 2 | 0 | 1 |
| 37 | R04C06 | 1 | 7 | 0 | 0 | 0 |
| 38 | R04C07 | 5 | 0 | 4 | 1 | 0 |
| 39 | R04C08 | 2 | 3 | 1 | 0 | 1 |
| 40 | R04C09 | 2 | 0 | 1 | 1 | 1 |
| 41 | R04C10 | 2 | 3 | 1 | 1 | 0 |
| 42 | R04C11 | 1 | 1 | 1 | 3 | 2 |
| 43 | R04C12 | 1 | 2 | 0 | 1 | 1 |
| 44 | R04C13 | 1 | 0 | 0 | 0 | 0 |
| 45 | R04C14 | 0 | 0 | 0 | 1 | 0 |
| 46 | R04C15 | 0 | 0 | 1 | 0 | 0 |
| 47 | R05C01 | 0 | 0 | 1 | 0 | 0 |
| 48 | R05C02 | 0 | 0 | 2 | 0 | 0 |
| 49 | R05C03 | 1 | 0 | 1 | 0 | 0 |
| 50 | R05C04 | 2 | 0 | 2 | 0 | 1 |
| 51 | R05C05 | 3 | 1 | 2 | 1 | 1 |
| 52 | R05C06 | 3 | 2 | 3 | 4 | 3 |
| 53 | R05C07 | 3 | 4 | 2 | 0 | 1 |
| 54 | R05C08 | 1 | 3 | 3 | 0 | 0 |
| 55 | R05C09 | 2 | 6 | 2 | 2 | 1 |
| 56 | R05C10 | 4 | 2 | 0 | 1 | 0 |
| 57 | R05C11 | 1 | 0 | 4 | 0 | 1 |
| 58 | R05C12 | 4 | 0 | 1 | 3 | 0 |
| 59 | R05C13 | 1 | 0 | 0 | 0 | 0 |
| 60 | R05C14 | 0 | 0 | 0 | 0 | 0 |
| 61 | R05C15 | 0 | 0 | 1 | 0 | 0 |
| 62 | R06C01 | 0 | 0 | 0 | 0 | 0 |
| 63 | R06C02 | 0 | 0 | 0 | 1 | 0 |
| 64 | R06C03 | 0 | 3 | 0 | 0 | 0 |
| 65 | R06C04 | 1 | 2 | 0 | 0 | 2 |
| 66 | R06C05 | 1 | 2 | 1 | 3 | 2 |
| 67 | R06C06 | 2 | 7 | 3 | 0 | 3 |
| 68 | R06C07 | 5 | 2 | 1 | 2 | 4 |
| 69 | R06C08 | 3 | 4 | 2 | 1 | 0 |
| 70 | R06C09 | 2 | 5 | 0 | 0 | 4 |
| 71 | R06C10 | 7 | 6 | 2 | 2 | 3 |
| 72 | R06C11 | 5 | 0 | 3 | 3 | 1 |
| 73 | R06C12 | 4 | 2 | 2 | 1 | 0 |
| 74 | R06C13 | 4 | 0 | 0 | 0 | 0 |
| 75 | R06C14 | 0 | 0 | 0 | 0 | 1 |
| 76 | R06C15 | 0 | 1 | 0 | 0 | 0 |
| 77 | R06C16 | 0 | 0 | 0 | 0 | 0 |
| 78 | R07C01 | 1 | 0 | 0 | 1 | 0 |
| 79 | R07C02 | 0 | 0 | 1 | 2 | 0 |
| 80 | R07C03 | 4 | 5 | 0 | 1 | 2 |
| 81 | R07C04 | 1 | 2 | 2 | 2 | 4 |
| 82 | R07C05 | 3 | 1 | 1 | 2 | 1 |
| 83 | R07C06 | 1 | 7 | 1 | 1 | 2 |
| 84 | R07C07 | 7 | 7 | 0 | 3 | 0 |
| 85 | R07C08 | 1 | 1 | 0 | 1 | 0 |
| 86 | R07C09 | 1 | 3 | 0 | 2 | 1 |
| 87 | R07C10 | 1 | 0 | 0 | 3 | 2 |
| 88 | R07C11 | 7 | 1 | 4 | 1 | 0 |
| 89 | R07C12 | 2 | 1 | 1 | 0 | 2 |
| 90 | R07C13 | 1 | 0 | 0 | 1 | 0 |
| 91 | R07C14 | 2 | 0 | 0 | 0 | 2 |
| 92 | R07C15 | 1 | 0 | 0 | 0 | 0 |
| 93 | R07C16 | 0 | 0 | 0 | 0 | 1 |
| 94 | R08C01 | 1 | 0 | 0 | 1 | 0 |
| 95 | R08C02 | 1 | 0 | 1 | 3 | 1 |
| 96 | R08C03 | 0 | 5 | 1 | 0 | 2 |
| 97 | R08C04 | 3 | 5 | 0 | 3 | 5 |
| 98 | R08C05 | 1 | 6 | 0 | 1 | 2 |
| 99 | R08C06 | 2 | 5 | 0 | 2 | 0 |
| 100 | R08C07 | 3 | 7 | 1 | 2 | 1 |
| 101 | R08C08 | 2 | 2 | 1 | 0 | 0 |
| 102 | R08C09 | 3 | 6 | 1 | 1 | 5 |
| 103 | R08C10 | 2 | 5 | 1 | 0 | 0 |
| 104 | R08C11 | 2 | 4 | 5 | 1 | 1 |
| 105 | R08C12 | 5 | 1 | 0 | 0 | 1 |
| 106 | R08C13 | 3 | 0 | 0 | 0 | 0 |
| 107 | R08C14 | 1 | 3 | 0 | 0 | 0 |
| 108 | R08C15 | 0 | 0 | 0 | 1 | 0 |
| 109 | R08C16 | 0 | 0 | 0 | 0 | 0 |
| 110 | R09C01 | 0 | 0 | 0 | 1 | 0 |
| 111 | R09C02 | 0 | 1 | 0 | 1 | 1 |
| 112 | R09C03 | 3 | 2 | 2 | 1 | 2 |
| 113 | R09C04 | 2 | 3 | 0 | 1 | 2 |
| 114 | R09C05 | 3 | 1 | 1 | 2 | 2 |
| 115 | R09C06 | 5 | 6 | 2 | 2 | 1 |
| 116 | R09C07 | 1 | 5 | 3 | 0 | 2 |
| 117 | R09C08 | 0 | 3 | 1 | 2 | 1 |
| 118 | R09C09 | 3 | 8 | 2 | 1 | 2 |
| 119 | R09C10 | 3 | 2 | 2 | 3 | 1 |
| 120 | R09C11 | 1 | 4 | 0 | 0 | 2 |
| 121 | R09C12 | 4 | 5 | 0 | 1 | 0 |
| 122 | R09C13 | 6 | 4 | 0 | 0 | 2 |
| 123 | R09C14 | 0 | 3 | 0 | 0 | 0 |
| 124 | R09C15 | 0 | 0 | 0 | 0 | 0 |
| 125 | R09C16 | 0 | 0 | 0 | 0 | 1 |
| 126 | R10C01 | 0 | 0 | 0 | 0 | 0 |
| 127 | R10C02 | 0 | 0 | 0 | 2 | 0 |
| 128 | R10C03 | 2 | 5 | 0 | 0 | 1 |
| 129 | R10C04 | 4 | 4 | 1 | 0 | 5 |
| 130 | R10C05 | 2 | 3 | 0 | 0 | 3 |
| 131 | R10C06 | 3 | 2 | 1 | 0 | 1 |
| 132 | R10C07 | 4 | 1 | 3 | 3 | 0 |
| 133 | R10C08 | 4 | 1 | 3 | 0 | 0 |
| 134 | R10C09 | 4 | 4 | 2 | 3 | 2 |
| 135 | R10C10 | 1 | 4 | 1 | 0 | 1 |
| 136 | R10C11 | 1 | 2 | 3 | 4 | 1 |
| 137 | R10C12 | 4 | 6 | 0 | 1 | 1 |
| 138 | R10C13 | 3 | 4 | 1 | 0 | 1 |
| 139 | R10C14 | 0 | 2 | 0 | 0 | 0 |
| 140 | R10C15 | 0 | 0 | 0 | 0 | 0 |
| 141 | R10C16 | 0 | 0 | 0 | 0 | 0 |
| 142 | R11C01 | 0 | 0 | 0 | 0 | 1 |
| 143 | R11C02 | 2 | 0 | 0 | 2 | 0 |
| 144 | R11C03 | 1 | 3 | 0 | 0 | 2 |
| 145 | R11C04 | 4 | 5 | 0 | 0 | 1 |
| 146 | R11C05 | 0 | 3 | 0 | 1 | 1 |
| 147 | R11C06 | 0 | 3 | 1 | 1 | 1 |
| 148 | R11C07 | 4 | 3 | 4 | 0 | 2 |
| 149 | R11C08 | 5 | 5 | 6 | 5 | 3 |
| 150 | R11C09 | 3 | 1 | 7 | 2 | 2 |
| 151 | R11C10 | 3 | 2 | 1 | 1 | 0 |
| 152 | R11C11 | 6 | 2 | 2 | 1 | 2 |
| 153 | R11C12 | 7 | 2 | 0 | 2 | 1 |
| 154 | R11C13 | 2 | 7 | 0 | 1 | 1 |
| 155 | R11C14 | 1 | 2 | 0 | 0 | 0 |
| 156 | R11C15 | 0 | 0 | 0 | 0 | 0 |
| 157 | R11C16 | 0 | 0 | 0 | 0 | 0 |
| 158 | R12C01 | 0 | 0 | 0 | 0 | 0 |
| 159 | R12C02 | 1 | 0 | 0 | 1 | 0 |
| 160 | R12C03 | 1 | 0 | 0 | 0 | 0 |
| 161 | R12C04 | 0 | 3 | 3 | 2 | 1 |
| 162 | R12C05 | 1 | 5 | 0 | 2 | 0 |
| 163 | R12C06 | 2 | 4 | 0 | 0 | 1 |
| 164 | R12C07 | 1 | 4 | 0 | 1 | 2 |
| 165 | R12C08 | 0 | 2 | 1 | 3 | 2 |
| 166 | R12C09 | 3 | 3 | 2 | 0 | 0 |
| 167 | R12C10 | 6 | 5 | 0 | 2 | 0 |
| 168 | R12C11 | 2 | 6 | 0 | 1 | 2 |
| 169 | R12C12 | 2 | 5 | 0 | 1 | 2 |
| 170 | R12C13 | 0 | 3 | 0 | 0 | 1 |
| 171 | R12C14 | 0 | 0 | 3 | 0 | 0 |
| 172 | R12C15 | 0 | 1 | 0 | 0 | 0 |
| 173 | R13C02 | 0 | 0 | 0 | 0 | 0 |
| 174 | R13C03 | 1 | 0 | 0 | 0 | 0 |
| 175 | R13C04 | 0 | 1 | 0 | 0 | 0 |
| 176 | R13C05 | 0 | 1 | 0 | 1 | 0 |
| 177 | R13C06 | 3 | 2 | 0 | 2 | 0 |
| 178 | R13C07 | 1 | 7 | 0 | 0 | 1 |
| 179 | R13C08 | 2 | 5 | 0 | 1 | 2 |
| 180 | R13C09 | 1 | 4 | 1 | 0 | 0 |
| 181 | R13C10 | 1 | 3 | 0 | 2 | 2 |
| 182 | R13C11 | 1 | 3 | 0 | 3 | 0 |
| 183 | R13C12 | 0 | 1 | 0 | 1 | 0 |
| 184 | R13C13 | 1 | 0 | 0 | 1 | 1 |
| 185 | R13C14 | 0 | 0 | 0 | 0 | 0 |
| 186 | R13C15 | 0 | 0 | 0 | 0 | 0 |
| 187 | R14C02 | 0 | 0 | 0 | 1 | 0 |
| 188 | R14C03 | 0 | 0 | 0 | 0 | 0 |
| 189 | R14C04 | 0 | 0 | 0 | 0 | 0 |
| 190 | R14C05 | 0 | 0 | 0 | 2 | 1 |
| 191 | R14C06 | 1 | 2 | 0 | 3 | 0 |
| 192 | R14C07 | 3 | 1 | 1 | 1 | 0 |
| 193 | R14C08 | 1 | 1 | 0 | 1 | 0 |
| 194 | R14C09 | 1 | 1 | 0 | 0 | 2 |
| 195 | R14C10 | 0 | 1 | 0 | 0 | 0 |
| 196 | R14C11 | 1 | 1 | 0 | 0 | 0 |
| 197 | R14C12 | 1 | 1 | 0 | 0 | 1 |
| 198 | R14C13 | 0 | 1 | 0 | 0 | 0 |
| 199 | R14C14 | 0 | 0 | 0 | 0 | 0 |
| 200 | R15C04 | 0 | 0 | 0 | 0 | 0 |
| 201 | R15C05 | 0 | 0 | 0 | 1 | 0 |
| 202 | R15C06 | 0 | 0 | 0 | 0 | 1 |
| 203 | R15C07 | 0 | 0 | 0 | 0 | 0 |
| 204 | R15C08 | 0 | 1 | 0 | 0 | 1 |
| 205 | R15C09 | 1 | 0 | 0 | 0 | 1 |
| 206 | R15C10 | 0 | 0 | 0 | 0 | 0 |
| 207 | R15C11 | 2 | 1 | 0 | 1 | 0 |
| 208 | R15C12 | 1 | 2 | 0 | 0 | 0 |
| 209 | R15C13 | 0 | 0 | 0 | 0 | 0 |
| 210 | R16C05 | 0 | 0 | 0 | 0 | 0 |
| 211 | R16C06 | 0 | 0 | 0 | 0 | 0 |
| 212 | R16C07 | 0 | 0 | 0 | 0 | 0 |
| 213 | R16C08 | 0 | 0 | 0 | 0 | 0 |
| 214 | R16C09 | 0 | 0 | 0 | 0 | 0 |
| 215 | R16C10 | 0 | 0 | 0 | 0 | 0 |
| 216 | R16C11 | 0 | 0 | 0 | 0 | 0 |
| 217 | R16C12 | 0 | 0 | 0 | 0 | 0 |
